# A Mendacitorium

**Published:** 1912-05

**Authors:** 


A MENDACITORIUM.—I propose this name for the "Hospital for Liars," or "Infirmary for the Mendacious," established at Lugville some years ago by an eccentric individual by the name of Jenks. He was an old bachelor,—a millionaire. He conceived the idea that lying is a moral disease, if not a distinct psychosis, and is amenable to treatment; and he proposed to treat it homeopathically —*similia similibus curantur*, just as its kindred disease, drunkenness—dipsomania—is sometimes treated, by giving the hair of the dog, you know, to cure the bite.

The source of the following remarkable account of a visit to this institution is an anonymous contribution to the *New York Times* some years ago:

We were met at the door of the Infirmary by a pleasant-faced gentleman, who spoke with a slight German accent, who introduced himself as the Assistant Superintendent. * * * We entered the office and registered our names in the "Visitors' Book."

"Our system," said the Assistant Superintendent, "is very simple. The theory of the institution is that the habit of mendacity, which, in many cases, becomes chronic, is a moral disease, like habitual inebriety, and that it can generally be cured. We take the liar who voluntarily submits himself to our treatment, and for six months we subject him to the forcing process. That is, we encourage him in lying, surround him with liars, his equals and superiors in skill, and cram him with falsehood until he is fairly saturated. By this time the reaction has set in, and the patient is usually starved for the truth. He is prepared to welcome the second course of treatment. For the next half-year the opposite method is pursued. The satiated and disgusted liar is surrounded by truthful attendants, encouraged to peruse veracious literature, and by force of lectures, example and moral influence brought to understand how much more creditable it is to say the thing which is, than the thing which is not. Then we send him back into the world; and I must say that cases of relapse are infrequent." * * *

"Somebody came in bringing a new patient. * * * The Assistant Superintendent invited me to follow him.

"I will show you how our patients live, and how they amuse themselves," he said. "We will go first, if you please, through the left wing, where the saturating process may be observed."

He led the way into a large room, comfortably furnished, and occupied by two dozen or more gentlemen, some reading, some writing, while others sat or stood in groups, engaged in animated talk. Indeed, if it had not been for the iron bars at the windows, I might have fancied myself in the lounging room of a respectable club. My guide stopped to speak to an inmate who was listlessly turning the leaves of a well-thumbed copy of Baron Munchausen, and left me near enough to one of the groups to overhear parts of the conversation:

"My rod creaked and bent double," a stout, red-faced gentleman was saying, "and the reel spun like a teetotum. I tell you, if Pierre Chaveau hadn't had the presence of mind to grip the most convenient part of my trousers with the boat hook, I should have been dragged into the lake in two seconds. Well, sir, we fought sixty-nine minutes, by actual time taking, and when I got him in, and had got him to the hotel, he tipped the scale,—the speckled beauty did,—at thirty-seven pounds and eleven-sixteenths, whether you believe it or not."

"Nonsense," said a quiet little gentleman who sat opposite. "That is impossible."

The first speaker looked flattered at this, and colored with pleasure. "Nevertheless," he retorted, "it's a fact, on my honor as a sportsman. Why do you say it's impossible?"

"Because," said the other, calmly, "it is an ascertained scientific fact, as every true fisherman in this room knows perfectly well, that there are no trout in Mooselemaguntìcook weighing under half a hundred."

"Certainly not," put in a third speaker. "The bottom of the lake is a sieve,—a sort of shistose sieve formation,—and all fish smaller than the fifty-pounders fall through."

"Why doesn't the water drop through, too?" asked the stout patient, in a triumphant tone.

"It used to," replied the quiet gentleman, gravely, "until the Legislature passed an act preventing it."

My guide rejoined me and we crossed to another room. "These sportsmen liars," he said, "are amongst the mildest and most easily cured cases that come here. We send them away in from six to nine weeks' time, with the habit broken up, and pledged not to fish or hunt any more. The man who lies about the fish he has caught, or about the intelligence of his red setter dog, is often, in all other respects, a trustworthy citizen. Yet such cases form nearly 40 per cent of all our patients."

"What are the most obstinate cases?" I asked.

"Undoubtedly those which you will see in the Travelers' and Politicians' wards of the Infirmary. The more benign cases, such as the fishermen liars, the society liars, the lady-killer or *bonnes fortunes* liars, the Rocky Mountain and frontier liars (excepting Texas cases), the railroad prospectus liars, the psychical research liars, and the miscellaneous liars of various classes, we permit, during the first stage of treatment, to mingle freely with each other. The effect is good. But we keep the Travelers and the Politicians strictly isolated."

He was about to conduct me out of the room, * * * when a detached phrase, uttered by a pompous gentleman, arrested my attention:

"Scipio Africanus once remarked to me—"

"There couldn't be a better example," said my guide, as we passed out of the room, "of what we call the forcing system in the treatment of mendacity. That patient came to us voluntarily about two months ago. The form of his disease is a common one. Perfectly truthful in all other respects, he can not resist the temptation to claim personal acquaintance and even intimacy with distinguished individuals. His friends laughed at him so much for this weakness that, when he heard of the establishment of the Infirmary, he came here, like a sensible man, and put himself under our care. He is doing splendidly. When he found that his reminiscences of Beaconfield and Bismarck and Victor Hugo created no sensation here, but were, on the contrary, at once matched and capped by still more remarkable experiences narrated by other inmates, he was at first a little staggered. But the habit is so strong, and the peculiar vanity that craves admiration on this score is so exacting, that he began to extend his acquaintance, gradually and cautiously, back into the past. Soon we find him giving reminiscences of Tallerand, of Thomas Jefferson and of Lord Cornwallis. Observe the psychological effect of our system. The ordinary checks on the performances of such a liar being removed, and, no doubt, suspicion, nor even wonder, being expressed at any of his anecdotes, he has gone back through Voltaire and William the Silent, to Charlemagne, and so on. There happens to be in the institution another patient with precisely the same trouble. They are, therefore, in active competition, and each serves to force the other back more rapidly. Not long ago I heard our friend in here describing one of Heliogabalus' banquets, which he had attended as an honored guest." "Why, I was there, too!" cried the other liar. "It was the night they gave us the boar's head stuffed with goose giblets, and that delicious dry Opimian muscadine."

"Well," I asked, "and what is your prognosis of this case?"

"Jut now the two personal reminiscence liars are driving each other back through ancient history at the rate of about three centuries a week. The flood isn't likely to stop them. Before long they will be matching reminiscences of the antediluvian patriarchs, and then they will bring up squarely on Adam. They can't go any further than Adam. By that time they will be ready for the truth-cure process; and after a few weeks spent in an atmosphere of strict veracity in the other wing of the Infirmary, they'll go out into the world again, perfectly cured, and much more useful citizens than before they came to us."

